# Implications of heart rate variability measured using wearable electrocardiogram devices in diagnosing Parkinson’s disease and its association with neuroimaging biomarkers: a case-control study

**DOI:** 10.3389/fnagi.2025.1530240

**Published:** 2025-05-12

**Authors:** Mincheol Park, Sung-Woo Kim, Jin Yong Hong, Sung Pil Cho, Junghwan Park, Erdenebayar Urtnasan, Min Seok Baek

**Affiliations:** ^1^Department of Neurology, Chung-Ang University College of Medicine, Seoul, Republic of Korea; ^2^Department of Neurology, Wonju Severance Christian Hospital, Yonsei University Wonju College of Medicine, Wonju, Republic of Korea; ^3^Research Institute of Metabolism and Inflammation, Yonsei University Wonju College of Medicine, Wonju, Republic of Korea; ^4^MEZOO Co., Ltd., Wonju, Republic of Korea; ^5^Artificial Intelligence Big Data Medical Center, Wonju College of Medicine, Yonsei University, Wonju, Republic of Korea; ^6^Yonsei Institute of AI Data Convergence Science, Yonsei University Mirae Campus, Wonju, Republic of Korea

**Keywords:** Parkinson’s disease, heart rate variability, electrocardiography, tremor, cerebellum

## Abstract

**Introduction:**

Heart rate variability (HRV) reflects cardiac autonomic regulation, and reduced HRV is associated with Parkinson’s disease (PD). However, studies regarding the implications of HRV measures for the clinical manifestations of PD have shown inconclusive results. We examined the relationship between HRV measures obtained via long-term monitoring using a wearable electrocardiogram (ECG) device and the diagnosis and clinical characteristics of PD.

**Methods:**

Seventeen controls and 20 patients with PD were prospectively enrolled. The HRV measures were recorded using a wearable ECG device for up to 72 h. Time- and frequency-domain measures were derived from the HRV analysis, and their association with PD diagnosis and clinical features was investigated. We investigated their association with neuroimaging biomarkers using magnetic resonance imaging to explore the underlying neural correlates.

**Results:**

The diagnosis of PD was associated with several HRV measures, including a decreased standard deviation of N-N intervals, standard deviation of all heart rates, and low-frequency (LF) power. Among these HRV measures, only LF power was associated with clinical features of PD. LF power was positively correlated with the tremor sub-score (*r* = 0.500; *p* = 0.035) and negatively associated with the left (*r* = −0.598; *p* = 0.024) and right (*r* = −0.693; *p* = 0.006) cerebellar hemispheres in patients with PD.

**Conclusion:**

Low-frequency power may be used as a biomarker for tremor-associated pathophysiology of PD. Moreover, a wearable ECG device with its capability for long-term monitoring might be a promising tool for diagnosing PD.

## 1 Introduction

Parkinson’s disease (PD) is the second most common neurodegenerative disorder, with various motor and non-motor symptoms throughout the disease course. Autonomic dysfunction is common in PD and may be present in the very early stages of PD, even before the onset of motor symptoms (Sharabi et al., 2021). Autonomic dysfunction can manifest as sympathetic, parasympathetic, and enteric nervous system dysfunctions, resulting in orthostatic hypotension, constipation, bladder dysfunction, and sexual dysfunction (Sharabi et al., 2021). Deposition of alpha-synuclein aggregates is particularly common in the cardiovascular autonomic nervous system because of the high density of sympathetic innervation in the heart (Goldstein and Sharabi, 2019). Cardiovascular autonomic dysfunction in PD may result in orthostatic hypotension, postprandial hypotension, supine hypertension, and non-dipping (Chen et al., 2020).

Regarding autonomic regulation of cardiac cycle, heart rate variability (HRV) refers to the variation in time between successive heartbeats and represents an index of the cardiac autonomic nervous system (Shaffer and Ginsberg, 2017). HRV is a simple and non-invasive tool for assessing cardiovascular autonomic regulation in diverse populations (Yoon et al., 2016, Alonso et al., 2015). Recently, wrist-worn trackers have become available for recording HRV, rendering HRV as a promising digital biomarker (Natarajan et al., 2020). Time- and frequency-domain measures and non-linear measurements can be derived from HRV analysis, and each measure is thought to be associated with its underpinning autonomic correlates (Shaffer and Ginsberg, 2017). Alterations in HRV have been linked to a variety of clinical conditions, including cardiovascular disease and metabolic disorders, and are increasingly recognized as reflective of neurodegenerative processes (Stuckey et al., 2014, Liu et al., 2022).

The implications of HRV in PD have been studied previously. Several cross-sectional and longitudinal studies have revealed decreased HRV measures in patients with PD compared with those in the healthy control and patients with essential tremor (Sorensen et al., 2013, Kallio et al., 2000, Yoon et al., 2016, Solla et al., 2015, Maetzler et al., 2015). Moreover, reduced HRV is observed in idiopathic rapid eye movement sleep behavior disorder, which could be a prodromal phase of PD (Valappil et al., 2010, Postuma et al., 2010). However, the association between HRV measures and the clinical manifestations of PD has been inconsistent. Some studies have revealed an association between HRV measures and clinical features, including hypokinesia or cognitive status (Terroba-Chambi et al., 2020, Haapaniemi et al., 2001), whereas others have failed to show an association between HRV measures and clinical manifestations (Maetzler et al., 2015, Mihci et al., 2006). These inconsistent findings may be attributed to the diverse study designs and HRV measurement methods. For example, short-term HRV recordings may not capture a sufficient duration to reflect certain daily activity patterns (Voss et al., 2013, Ruangsuphaphichat et al., 2023), and hospital-based electrocardiogram (ECG) monitoring systems, such as polysomnography-embedded ECG devices, have limited feasibility for patients and only provide data for a specific time of the day (Kwon et al., 2023). A recent study revealed an association between striatal dopaminergic availability and HRV measures (Kitagawa et al., 2021); however, the neural correlates of reduced HRV in patients with PD have not been clearly elucidated.

In this study, we examined the relationship between HRV measures obtained via long-term monitoring using a wearable ECG device and the clinical characteristics of PD. The present study leverages long-term HRV monitoring using a wearable ECG device in conjunction with advanced neuroimaging to (1) delineate the HRV parameters that distinguish PD patients from controls, (2) explore the association between HRV metrics and clinical features of PD, and (3) identify the neural substrates underlying these autonomic alterations. By integrating these methodologies, our work aims to advance the understanding of autonomic dysfunction in PD and to establish novel biomarkers that may facilitate earlier diagnosis of PD.

## 2 Materials and methods

### 2.1 Participants

We prospectively enrolled 20 patients with PD and 17 controls who visited the Memory Disorder Clinic of Wonju Severance Christian Hospital between May 2022 and October 2022. PD was diagnosed according to the clinical criteria of the United Kindgom Brain Bank (Gibb and Lees, 1988) and the presence of appropriate dopamine transporter defects on ^18^F-N-(3-fluoropropyl)-2β-carboxymethoxy-3β-(4-iodophenyl) nortropane positron emission tomography. Control group was defined as those who showed no focal neurological symptoms and exhibited no signs of parkinsonism on neurological examinations. Individuals with a medical history of cerebrovascular disease or cardiovascular disease, and dementia, current psychosis, or mental illness were excluded. All patients underwent brain T1-weighted magnetic resonance (MR) images for volumetric analysis and the Korean version of the Montreal Cognitive Assessment for cognitive assessment. Motor and non-motor symptoms were assessed using the Movement Disorder Society-Unified Parkinson’s Disease Rating Scale scores (MDS-UPDRS). The sub-scores for tremor (items 3.15, 3.16, 3.17, and 3.18), rigidity (item 3.3), bradykinesia (items 3.4, 3.5, 3.6, 3.7, 3.8, 3.9, and 3.14), and gait and postural instability (items 3.10, 3.11, and 3.12) were obtained from the MDS-UPDRS part III (Hong et al., 2022). The clinical diagnoses of the participants were established through mutual agreement between two neurology specialists (MSB and JYH).

This study was approved by the Institutional Review Board of Wonju Severance Hospital (Ref# CR221020), and the research protocol was aligned with the principles of the Declaration of Helsinki and its subsequent revisions.

### 2.2 Wearable device, data measurement, and analysis

The Hicardi Plus (MEZOO Co., Ltd., Wonju-si, Gangwon-do, Korea) is a lightweight (18 g), compact (60 × 40 × 10 mm), wireless, and wearable adhesive monitoring device certified as a medical device by the Ministry of Food and Drug Safety of Korea. It monitors and records single-lead ECGs, respiratory rates, skin surface temperatures, and activities (Supplementary Figure 1). The device consists of a reusable sensor module and disposable adhesive patch that houses a two-point ECG electrode. It attaches to the chest and can continuously measure signals for up to 72 h. To ensure anonymization, patient information is not recorded on the wearable device. The ECG signal is recorded at a rate of 250 samples/s with16-bit resolution.

The data from the wearable device were transferred in packets through Bluetooth Low Energy (BLE) to a mobile gateway implemented as a smartphone application, which then transmitted the data packets to a cloud-based monitoring server via a cellular or home WiFi network. Because BLE communication has a short transmission distance, participants were instructed to keep the smartphone with the mobile gateway installed nearby (within 1.5 m) during the measurement period to minimize potential data loss. The ECG signals and the abovementioned data were continuously recorded during daily living and analyzed after data collection via monitoring server.

In this study, ECG records averaging 53.4 h per patient were obtained during daily living, including both wakefulness and sleep periods. Each ECG record data was measured during daily living rather than in a controlled environment such as a hospital ward; it may contain data losses resulting from various communication errors, including BLE connectivity disruptions and errors in WiFi or cellular transmission. Thus, prior to analysis, each record is required to undergo the identification and exclusion process of segments affected by communication-related data losses or signal disruptions. Subsequently, preprocessing is conducted, including noise filtering and artifact removal, followed by extraction of valid R-peaks. Finally, HRV parameters are calculated from the validated ECG data segments for further analysis.

Although each ECG record was recorded for durations exceeding 24 h, ultra-low frequency (ULF), a frequency-domain parameter of HRV, was not computed due to intermittent data losses resulting from communication errors. The ULF parameter requires continuous and uninterrupted long-term ECG data to reliably detect very low-frequency physiological rhythms; therefore, even small data gaps significantly impair the accuracy of ULF calculations. Instead, ECG recordings were segmented into consecutive 5 min intervals, and short-term HRV parameters were calculated from these intervals to ensure analytical reliability and validity.

To conduct the aforementioned procedure, each ECG record was first divided into consecutive 5 min segments, and each segment was examined for data loss due to communication errors. When a data packet is generated by the wearable ECG patch, an identification number is assigned that increments sequentially by one; thus, packet loss can be identified by checking the continuity of these identification numbers. Next, the occurrences of electrode detachment and intermittent electrode contact—referred to as “lead-off,” “lead-fail,” or “lead-fault”—which can cause additional data loss, were identified. A lead-off condition is characterized by a drop in signal on one or more of the ECG leads or electrodes. During the ECG recording, sudden movements by the participant or scratching around the patch with their hand can cause a momentary lead-off. The wearable ECG patch has an analog front end (AFE) that automatically detects lead off and is set to output a zero signal when lead off is detected. Most of the lead-offs were mainly occurred in the process of attaching the electrode at the beginning of the measurement and detaching the electrode after the measurement.

Through the above process, each segment was validated, and subsequently, R-peaks were detected within each valid segment. The R-peaks were detected using the geometric angle between two consecutive samples of the ECG signal (Song et al., 2015). The R-peaks detection algorithm showed 99.95% of the sensitivity, 99.95% of the positive predictivity, and 0.10% of the detection fail rate on the four different databases in the previous study was adopted and modified to be suitable for wearable ECG signal. This method utilizes a simple adaptive thresholding technique and a geometric angle which is formed by two consecutive samples of ECG. By converting the amplitude change of the ECG signal into angle rather than size, the method has the advantage of accurately detecting R peaks without being affected by irregular QRS group shapes, sudden changes in size or interval, or sudden changes in QRS group morphology such as ectopic beats. Due to the non-linear characteristics of the angle, is robust and insensitive to the variation of the amplitude and morphology of the QRS complex and baseline drift as well as abrupt change. To enhance the performance of R-peak detection, an adaptive filter for reducing the baseline wander was added to the previous method before calculating the geometric angle. And 64*^th^* order finite impulse response low pass filter with a cut-off frequency of 25 Hz was used to eliminate high-frequency noise. The low-pass filtered signal was utilized exclusively for angle-based detection to identify candidate R-peak regions. The final R-peak positions were determined by locating the local maxima on the baseline-corrected, but unfiltered ECG signal within a narrow window around each detected region. This approach preserves the morphological integrity of the QRS complex while maintaining the robustness of R-peak detection, thereby minimizing the risk of false detections induced by noise.

Heart rate variability analysis in both the frequency and time domains of wearable ECG recordings was conducted according to international guidelines (Task Force of the European Society of Cardiology the North American Society of Pacing Electrophysiology, 1996) using the detected R-peaks. In this study, the following steps have been performed for HRV analysis. First, the detected R-peaks were used to generate the R-R interval time series. And the N-N (Normal-to-Normal) intervals (that is all intervals between consecutive sinus-originated QRS complexes) were obtained by removing the abnormal intervals caused by ectopic beats, arrhythmic events, missing data, and noise, intervals below 80% or above 120% of the average of the last six intervals were excluded. Although the international guidelines suggest that excluding R-R intervals based solely on ± 20% deviation may not be optimal, recommending instead manual editing of R-R intervals, manual verification of every beat was impractical given the long-term nature of our data. Therefore, we applied an 80%–120% interval criterion to automatically exclude beats likely to be missed by manual review, except in cases of clear arrhythmias.

The time-domain parameters were calculated from the N-N interval time series. The mean heart rate (mean HR) of each 5 min ECG segment was calculated by dividing 60 by the mean N-N interval (in seconds). Similarly, the maximum heart rate (HR_*max*_) and minimum heart rate (HR_*min*_) were obtained by dividing 60 by the shortest and longest N-N intervals (in seconds) within each segment, respectively. The difference between HR_*max*_ and HR_*min*_ (HR_*max*_-HR_*min*_) was regarded a time-domain parameter. The root mean square of successive differences (RMSSD) was calculated by taking the square root of the mean squared differences between adjacent N-N intervals with each 5 min ECG segment. Also, the standard deviation of the N-N intervals (SDNN), standard deviation of all heart rates (STD), and percentage of adjacent N-N intervals that differed by > 50 ms (pNN50) were calculated.

Next, interpolation and resampling were performed to mitigate discontinuities caused by the removal of abnormal intervals and to convert the irregularly spaced N-N intervals into evenly spaced time series data, ensuring compatibility with frequency-domain HRV analysis. In this study, linear interpolation was applied for computational simplicity, followed by resampling at 4 Hz to meet the requirements for spectral analysis and then detrended by eliminating the linear trends. After detrending, the power spectral density for the evenly spaced N-N interval time series was estimated using a periodogram based on the fast Fourier transform. In the frequency-domain analysis, we examined low frequency (LF, 0.04–0.15 Hz), which is an index of both sympathetic and parasympathetic activity, and high frequency (HF, 0.15–0.4 Hz), which primarily represents parasympathetic activity to the sinus node. Very low frequency (VLF, 0.003–0.04 Hz), which partially reflects thermoregulatory mechanisms, fluctuations in renin–angiotensin system activity, and the function of peripheral chemoreceptors, and the LF/HF ratio, which indicates sympathovagal balance, were obtained. Specifically, each 5 min segment of the interpolated N-N interval time series (resampled at 4 Hz) was multiplied by a Hann window, and the FFT-based periodogram was applied directly to the entire segment. Each segment yielded a single PSD estimate, from which LF, HF, VLF power, and LF/HF ratios were calculated. The PSD estimates from each segment were then averaged to obtain representative PSD values.

The ECG signal preprocessing and HRV analysis, including signal filtering, R-peak detection, interpolation, resampling, and spectral analysis, were conducted using MATLAB (version 2023a; MathWorks, Natick, MA, United States).

### 2.3 MR image acquisition and preprocessing

Three-dimensional T1-weighted MR images were obtained using magnetization-prepared rapid acquisition gradient-echo sequences with the following parameters: slice thickness of 0.9 mm, pixel size of 0.4492 × 0.4492 mm, repetition time of 2,000 ms, echo time of 2.43 ms, flip angle of 9-degree, and matrix size of 512 × 512 pixels. Images were reconstructed to 512 × 512 pixels over a 256 mm field of view. The MR images were processed using FreeSurfer v.7.4.0^1^ to extract the bilateral thalamus and cerebellar gray matter regions and estimate their volumes. The FreeSurfer pipeline was used to estimate the intracranial volume (ICV) of each participant.

### 2.4 Statistical analysis

Statistical analyses were performed using the Statistical Package for the Social Sciences software (version 26.0; IBM Corp., Armonk, NY, United States). Comparisons of baseline characteristics and HRV measures between the control and PD groups were performed using the Mann–Whitney U test and Fisher’s exact test for continuous and categorical variables, respectively. The association between HRV measures and the diagnosis of PD was investigated using logistic regression. In separate models for each HRV metric, the diagnosis of PD was used as the dependent variable, each HRV measure as the independent variable, and age and sex as covariates. The sensitivity and specificity for differentiating the PD group from the control group were assessed using the receiver operating characteristic (ROC) curve analysis, with age and sex as covariates. The association between HRV measures and clinical metrics was analyzed using Pearson partial correlation, with HRV measure as an independent variable and age and sex as covariates among patients with PD. The association between HRV measure and the volume of the thalamus and cerebellar cortex was analyzed using Pearson partial correlation, with HRV measure as an independent variable and age, sex, and ICV as covariates. Owing to the small sample size, correction for multiple comparisons was not performed in this study. For all analyses, statistical significance was set at a *p*-value of < 0.05.

### 2.5 Data availability

The anonymized data supporting the findings of this study are available upon request from the corresponding authors. The data are not publicly available because of privacy and ethical restrictions.

## 3 Results

### 3.1 Baseline characteristics of study participants

The demographic data, clinical characteristics, and HRV measures of the study participants are summarized in Table 1. Age, sex, education, and history of hypertension, diabetes mellitus (DM), and dyslipidemia were comparable between the control and PD groups. Patients with PD had higher UPDRS I (5.71 ± 4.54 vs. 14.15 ± 9.07, *p* = 0.001), UPDRS II (1.71 ± 3.10 vs. 15.90 ± 11.65, *p* < 0.001), UPDRS III (0.94 ± 1.52 vs. 25.70 ± 16.16, *p* < 0.001), and motor sub-scores and lower Mini-Mental State Examination score (26.53 ± 2.72 vs. 21.61 ± 5.77, *p* = 0.003) than the control group. Regarding HRV measures, the PD group had lower HR_*max*_-HR_*min*_ (16.57 ± 3.31 vs. 11.33 ± 4.02, *p* < 0.001), SDNN (21.45 ± 7.84 vs. 13.23 ± 4.26, *p* < 0.001), STD (1.77 ± 0.66 vs. 1.18 ± 0.38, *p* = 0.002), pNN50 (14.83 ± 14.12 vs. 6.98 ± 8.53, *p* = 0.030), RMSSD (24.89 ± 10.68 vs. 16.19 ± 6.30, *p* = 0.004), VLF (0.91 ± 2.08 vs. 0.02 ± 0.04, *p* = 0.001), LF (0.12 ± 0.23 vs. 0.03 ± 0.02, *p* = 0.001), and HF (0.13 ± 0.25 vs. 0.03 ± 0.02, *p* = 0.004) power. The LF/HF ratios were comparable between the PD and control groups.

**TABLE 1:** Baseline characteristics of study participants.

Variables	Control	PD	*P*-value
Number	17	20	
Age	71.59 ± 8.80	71.45 ± 6.59	1.000
Sex, female	7 (41.2)	12 (60.0)	0.330
Symptom duration, years	NA	2.99 ± 2.40	NA
Education	11.06 ± 5.10	7.78 ± 4.79	0.080
Hypertension	9 (52.9)	9 (45.0)	0.746
Diabetes mellitus	3 (17.6)	4 (20.0)	1.000
Dyslipidemia	11 (64.7)	6 (30.0)	0.050
UPDRS I	5.71 ± 4.54	14.15 ± 9.07	0.001
UPDRS II	1.71 ± 3.10	15.90 ± 11.65	< 0.001
UPDRS III	0.94 ± 1.52	25.70 ± 16.16	< 0.001
Tremor sub-score	0.35 ± 0.86	2.35 ± 1.98	< 0.001
Rigidity sub-score	0.0	5.35 ± 4.16	< 0.001
Bradykinesia sub-score	0.29 ± 0.69	11.40 ± 7.18	< 0.001
Gait sub-score	0.06 ± 0.24	3.35 ± 3.05	< 0.001
MMSE	26.53 ± 2.72	21.61 ± 5.77	0.003
**HRV measures**			
**Time-domain measures**			
Mean HR	68.92 ± 9.26	73.35 ± 8.34	0.135
HR_max_–HR_min_	16.57 ± 3.31	11.33 ± 4.02	< 0.001
SDNN, ms	21.45 ± 7.84	13.23 ± 4.26	< 0.001
STD	1.77 ± 0.66	1.18 ± 0.38	0.002
pNN50, %	14.83 ± 14.12	6.98 ± 8.53	0.030
RMSSD	24.89 ± 10.68	16.19 ± 6.30	0.004
**Frequency-domain measures**			
VLF power, ms^2^	0.91 ± 2.08	0.02 ± 0.04	0.001
LF power, ms^2^	0.12 ± 0.23	0.03 ± 0.02	0.001
HF power, ms^2^	0.13 ± 0.25	0.03 ± 0.02	0.004
LF/HF ratio	2.13 ± 0.91	1.68 ± 0.77	0.158

The results are represented in mean ± standard deviation or n (%) using the Mann–Whitney U test and Fisher’s exact test for continuous and categorical variables, respectively. PD, Parkinson’s disease; UPDRS, Unified Parkinson’s Disease Rating Scale; MMSE, Mini-Mental State Examination; HRV, heart rate variability; HR, heart rate; HR_max_–HR_min_, difference between maximal heart rate and minimal heart rate; SDNN, standard deviation of NN intervals; STD, standard deviation of all heart rates; pNN50, percentage of successive interbeat intervals that differ by > 50 ms; RMSSD, root mean square successive difference; VLF, very low frequency; LF, low frequency; HF, high frequency; NA, not applicable.

### 3.2 HRV measures associated with PD diagnosis

The independent risks of HRV measures for the diagnosis of PD are shown in Table 2. Among the time-domain measures, high HR_*max*_–HR_*min*_ [odds ratio (OR), 0.579; 95% confidence interval (CI), 0.405–0.828; *p* = 0.003], SDNN (OR, 0.725; 95% CI, 0.578–0.909; *p* = 0.005), STD (OR, 0.044; 95% CI, 0.004–0.559; *p* = 0.016), and RMSSD (OR, 0.858; 95% CI, 0.755–0.975; *p* = 0.019) were associated with a low risk of PD, independent of age and sex, whereas pNN50 showed a trend of negative association with the risk of PD (OR, 0.784; 95% CI, 0.614–1.001; *p* = 0.051). Among the frequency-domain measures, high LF power was associated with a low risk of PD, independent of age and sex (OR, 3.42 × 10^–27^; 95% CI, 0.000–0.000; *p* = 0.010).

**TABLE 2:** Association between heart rate variability measures and Parkinson’s disease diagnosis.

HRV measures	OR	95% CI	*P*-value
**Time-domain measures**			
Mean HR	1.059	0.973–1.153	0.184
HR_max_–HR_min_	0.579	0.405–0.828	0.003
SDNN, ms	0.725	0.578–0.909	0.005
STD	0.044	0.004–0.559	0.016
pNN50, %	0.784	0.614–1.001	0.051
RMSSD	0.858	0.755–0.975	0.019
**Frequency-domain measures**			
VLF power, ms^2^	1.92 × 10^–5^	0.000–3.140	0.076
LF power, ms^2^	3.420 × 10^–27^	0.000–0.000	0.010
HF power, ms^2^	5.345 × 10^–16^	0.000–24.077	0.072
LF/HF ratio	0.516	0.202–1.316	0.166

The results are derived from individual logistic regression models, with heart rate variability measures as independent variables and age and sex as covariates. HR, heart rate; HR_max_–HR_min_, difference between maximal heart rate and minimal heart rate; SDNN, standard deviation of NN intervals; STD, standard deviation of all heart rates; pNN50, percentage of successive interbeat intervals that differ by > 50 ms; RMSSD, root mean square successive difference; VLF, very low-frequency power; LF, low-frequency power; HF, high-frequency power; OR, odds ratio; CI, confidence interval.

### 3.3 ROC curve analyses

**FIGURE 1:**
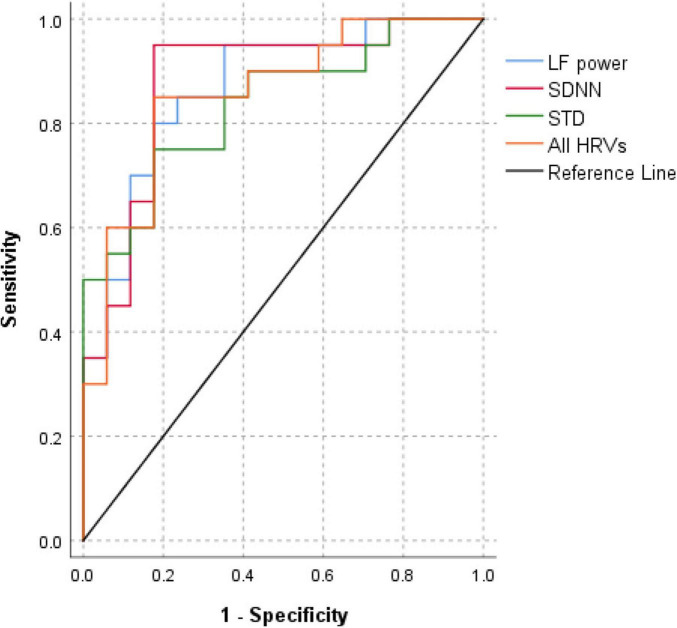
Comparison of receiver operating characteristic curves for heart rate variability measures in differentiating the Parkinson’s disease group from the control group. Receiver operating characteristic area under the curve (AUC) analysis reveals that HR_max_–HR_min_, SDNN, STD, RMSSD, LF power, and integrating all HRV metrics led to good diagnostic accuracy [AUC for HR_max_–HR_min_, 0.885; 95% confidence interval (CI), 0.768–1.003; AUC for SDNN, 0.879; 95% CI, 0.760–0.999; AUC for STD, 0.835; 95% CI, 0.706–0.964; AUC for RMSSD, 0.821; 95% CI, 0.675–0.966; AUC for LF power, 0.868; 95% CI, 0.750–0.985; AUC for all HRV metrics including HR_max_–HR_min_, SDNN, STD, RMSSD, and LF power, 0.935; 95% CI, 0.851–1.020]. HR_max_–HR_min_, difference between maximal heart rate and minimal heart rate; LF, low frequency; RMSSD, root mean square successive difference; SDNN, standard deviation of the N-N intervals; STD, standard deviation of all heart rates; HRV, heart rate variability.

By combining HRV measures that were significantly correlated with PD risk with age and sex, ROC curve analyses showed that these HRV measures had good diagnostic accuracy [area under the curve (AUC) for HR_*max*_–HR_*min*_, 0.885; 95% CI, 0.768–1.003; AUC for SDNN, 0.879; 95% CI, 0.760–0.999; AUC for STD, 0.835; 95% CI, 0.706–0.964; AUC for RMSSD, 0.821; 95% CI, 0.675–0.966; AUC for LF power, 0.868; 95% CI, 0.750–0.985; AUC for all HRVs, 0.935; 95% CI, 0.851–1.020; Figure 1]. Pairwise comparisons of the ROC curves did not reveal any significant differences (Supplementary Table 1).

### 3.4 Association between HRV measures and clinical manifestations in PD

The association between HRV measures and the clinical features of patients with PD is shown in Table 3. Among the HRV measures that showed a significant association with PD diagnosis, RMSSD and LF power was positively associated with the bradykinesia subscore (*r* = 0.495, *p* = 0.037) tremor sub-score (*r* = 0.500, *p* = 0.035), respectively, whereas HR_max_–HR_min_, SDNN and STD were not associated with any clinical features of PD.

**TABLE 3:** Association between heart rate variability measures and clinical features in Parkinson’s disease.

Clinical variables	SDNN		STD		HR_max_–HR_min_		RMSSD		LF power	
	***r***	***P***	***r***	***P***	***r***	***P***	***r***	***P***	***r***	***P***
UPDRS I	0.302	0.223	−0.014	0.956	−0.277	0.265	0.326	0.186	0.377	0.123
UPDRS II	0.313	0.206	−0.101	0.691	−0.322	0.193	0.299	0.228	0.385	0.115
UPDRS III	0.394	0.106	0.155	0.539	−0.129	0.611	0.435	0.071	0.175	0.487
UPDRS III Tremor	0.340	0.167	0.162	0.520	0.031	0.902	0.254	0.308	0.500	0.035
UPDRS III Rigidity	0.340	0.168	0.312	0.208	0.013	0.961	0.406	0.094	−0.001	0.996
UPDRS III Bradykinesia	0.438	0.069	0.172	0.495	−0.050	0.845	0.495	0.037	0.168	0.505
UPDRS3 III Gait	0.313	0.205	0.017	0.947	−0.233	0.351	0.269	0.281	0.375	0.125
MMSE	0.167	0.537	0.143	0.596	−0.092	0.736	0.200	0.460	0.011	0.968

The results are derived from Pearson’s partial correlation analysis between clinical features and heart rate variability measures, with age and sex as covariates. UPDRS, Unified Parkinson’s Disease Rating Scale; MMSE, Mini-Mental State Examination; SDNN, standard deviation of NN intervals; STD, standard deviation of all heart rates; HR_max_–HR_min_, difference between maximal heart rate and minimal heart rate; RMSSD, root mean square successive difference; LF, low frequency.

### 3.5 Association between LF power and tremor-associated brain regions

**FIGURE 2:**
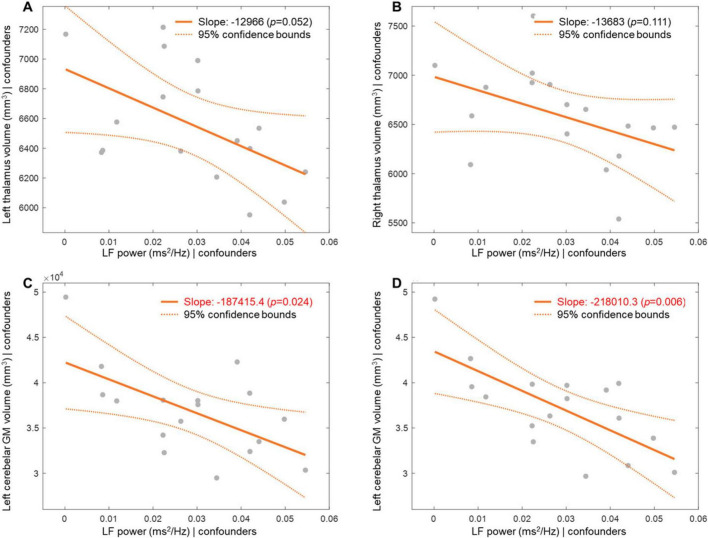
Association between low frequency power and tremor-associated regional volume. Pearson partial correlation shows association between LF power and GM volume of the left thalamus **(A)**, right thalamus **(B)**, left cerebellum **(C)**, and right cerebellum **(D)**. Bold orange lines represent regression lines from multiple linear regression models of LF power on GM volumes, adjusted for age, sex, and intracranial volume, with dashed lines indicating their 95% confidence bounds. GM, gray matter; LF, low frequency.

The association between LF power and volume of tremor-associated regions in the brain is illustrated in Figure 2 and Supplementary Table 2. Higher LF power was associated with lower cerebellar volume in both the left (*r* = −0.598, *p* = 0.024) and right (*r* = −0.693, *p* = 0.006) hemispheres after considering age, sex, and ICV as covariates. Higher LF power had a tendency of negative correlation with the volumes of the left (*p* = 0.052) and right (*p* = 0.111) thalami.

## 4 Discussion

In this study, we investigated the clinical implications and associated neural correlates of HRV in patients with PD, using a wearable ECG device. Our findings were as follows: (1) the diagnosis of PD was associated with several HRV measures, including low LF power, with good diagnostic accuracy; (2) LF power was positively associated with tremor sub-scores in patients with PD; and (3) LF power was negatively associated with cerebellar cortical volume in patients with PD. Taken together, our results suggest that LF power in HRV is associated with tremor-associated pathophysiological processes in PD.

Several studies have compared HRV measures to distinguish PD from other conditions, including essential tremor (ET), atypical parkinsonism, and HCs. Patients with PD had lower HRV measures than those with ET (Yoon et al., 2016), and several studies have shown that patients with PD had lower HRV measures than controls (Haapaniemi et al., 2001, Mihci et al., 2006, Solla et al., 2015, Maetzler et al., 2015). Even patients with idiopathic rapid eye movement sleep behavior disorder, which is often regarded as the prodromal phase of PD, had reduced HRV compared with the control population (Postuma et al., 2010, Valappil et al., 2010). Considering the early involvement of the autonomic nervous system in the pathological stages of PD (Braak and Del Tredici, 2008) and the incidental finding of Lewy pathology in the autonomic nervous system of patients without any clinical symptoms (Iwanaga et al., 1999), HRV measures may be used as sensitive biomarkers for the diagnosis of PD. In our study, the comparison of HRV measures showed reduced SDNN, STD, pNN50, VLF power, LF power, and HF power in patients with PD than in controls. However, as age and sex could influence HRV measures in the general population (Shaffer and Ginsberg, 2017), a comparison of HRV between groups should consider age and sex. When age and sex were considered as covariates, SDNN, STD, HR_max_–HR_min_, RMSSD, and LF power were associated with PD diagnosis. However, among these HRV measures, only LF power showed a significant correlation with the clinical manifestations of PD, especially the tremor sub-score, whereas other HRV measures did not show a significant correlation with the clinical manifestations of PD. This result indicates that among the HRV measures, LF power may reflect tremor-associated pathophysiology in PD. Previous studies have revealed a variable association between LF power and the clinical manifestations of PD. Studies have shown that LF power distinguished patients with PD from controls (Yoon et al., 2016) and that patients with PD with the tremor-dominant subtype had a higher LF power than those with the akinetic-rigid subtype (Solla et al., 2015), which is in line with our study. In terms of the autonomic basis of LF power, a previous study has shown that LF power is associated with sympathetic function (Malik et al., 1996), whereas another study has shown that LF power reflects parasympathetic function (Reyes del Paso et al., 2013). Moreover, another study has shown that LF power is correlated with baroreceptor activity (Goldstein et al., 2011). In addition, whether preganglionic or postganglionic fibers contribute to LF power has not been fully investigated. Therefore, the underlying physiology of LF power has not been fully elucidated. However, in PD, although both preganglionic and postganglionic fibers are affected, postganglionic fibers are more frequently and confluently involved in the early stage of the disease, compared to multiple system atrophy (Druschky et al., 2000). Moreover, the sympathetic cardiac nervous system is more profoundly involved than the parasympathetic cardiac innervation in early PD (Sorensen et al., 2013). Therefore, cardiac postganglionic sympathetic dysfunction may be associated with reduced LF power in patients with PD. However, future studies dissecting parasympathetic from sympathetic and preganglionic from postganglionic pathophysiology are warranted to unveil the mechanisms underlying reduced LF power in PD.

In PD, tremors are associated with multiple neurotransmitter systems other than dopamine alone, including serotonin, noradrenaline, and acetylcholine (Dirkx and Bologna, 2022). In our study, higher LF power was associated with a higher tremor score, indicating a common pathophysiological process between LF power and tremor in PD. A prior study revealed the relative preservation of cardiac metaiodobenzylguanidine scans in tremor-dominant PD compared with that in akinetic-rigid PD (Spiegel et al., 2007). Moreover, a low proportion of patients with PD with multiple-domain autonomic dysfunction had the tremor-dominant subtype (Zhou et al., 2021), and patients with tremor-dominant PD had lower SCOPA-AUT scores than those with the postural instability and gait disorders subtype (Malek et al., 2017). In addition, the noradrenergic subtype of PD is characterized by akinetic-rigid postural instability and gait disturbance motor manifestation rather than tremor (Ray Chaudhuri et al., 2023). These findings are consistent with our findings showing a positive association between LF power and tremor sub-scores. Therefore, cardiac autonomic preservation and tremor-dominant phenotypes may be associated with PD. However, the pathophysiological connection between the autonomic nervous system and tremor severity in PD has not been clearly elucidated. Preserved adrenergic circuitry is associated with tremor (Paulus and Jellinger, 1991), and LF power is associated with both the sympathetic and parasympathetic nervous systems (Shaffer and Ginsberg, 2017). Therefore, high LF power may reflect the relative preservation of the adrenergic system, which in turn correlates with a higher tremor score. However, as we could not directly investigate the adrenergic function nor noradrenaline-associated brain structures in our study, this assumption should be investigated in future studies.

In addition to nigrostriatal dopaminergic deficiency, dysfunction of the cerebellothalamocortical circuit is crucial in the pathophysiology of tremor in PD (Dirkx and Bologna, 2022). A recent study revealed differential patterns of cerebellar atrophy and cerebellar cortical volume in patients with PD, with and without tremor (Piccinin et al., 2017). As high LF power correlated with high tremor scores in our study, we investigated whether tremor-associated regions were associated with LF power. Interestingly, the bilateral cerebellar cortical volume was negatively associated with LF power. This is in line with the finding that LF power was positively correlated with the tremor sub-score, in that decreased cerebellar cortical volume could be associated with higher tremor sub-scores. On the other hand, cerebellum participates in cardiovascular autonomic control. The cerebellum has been recognized as a key region of the central autonomic network, (Sklerov et al., 2019) specifically impacting cardiovascular function by regulating blood pressure and HRV (Baker et al., 2019, Napadow et al., 2008, Baker and Kimpinski, 2020). High LF power could be associated with a preserved adrenergic nervous system and reduced cerebellar volume, both of which are associated with tremor pathogenesis as well as with a certain phenotype that represents the motor presentation of tremor in PD. However, the association between LF power, tremor sub-score, and cerebellar cortical volume might be complicated, and because of the small number of study participants, we did not further investigate the relationship between LF power, tremor sub-score, and cerebellar cortical volume in this study. Future studies on cardiac autonomic dysfunction, tremor-associated motor phenotypes, and the cerebellum are warranted.

In summary, in our study, patients with PD had lower LF power than controls, and lower LF power was associated with PD diagnosis when considering age and sex. Moreover, LF power was positively correlated with the tremor sub-score and negatively correlated with cerebellar cortical volume. As the cerebellum may play a role in both the tremor-associated network and the central autonomic network, these results imply that LF power may be utilized as a biomarker reflecting tremor-associated pathophysiological processes in PD.

Our study has some limitations. First, the relatively small number of study participants may limit the generalizability of our findings. Furthermore, the odds ratio for PD diagnosis associated with LF power was modest, which may constrain the clinical implications of LF power as a diagnostic marker for PD. However, given the small unit scale of LF power and the limited sample size, our study emphasizes the significant associations observed between LF power and PD diagnosis, tremor severity, and cerebellar cortical volume. These findings suggest that LF power may serve as a potential biomarker reflecting the function of the adrenergic nervous system, which in turn might be linked to neurophysiological correlates of tremor in PD. Therefore, the association between reduced HRV and tremor-associated pathophysiological findings, including cerebellar dysfunction, needs to be validated in a larger population. Second, as LF power was associated with tremor sub-scores among the clinical manifestations of PD, we investigated the association between LF power and tremor-associated structures, including the thalamus and cerebellar cortex. Therefore, the association between HRV measures and other brain structures including the central autonomic network or tremor-associated structures such as the neocortex, deep gray matter, and brainstem, should be investigated in the future. Third, other comorbidities that may have affected HRV were not considered in this study. Altered HRV is associated with diverse pathological and physiological conditions (e.g., DM, heart failure, structural heart disease, and psychiatric disorders) (Sammito and Böckelmann, 2016), and our study participants included those with DM. As the number of study participants was small and the frequency of DM was comparable between the control and PD group, we included diabetic patients in order to maximize the study participants. However, as cardiac autonomic nervous system dysfunction and HRV alteration are common in DM (Benichou et al., 2018, Dimitropoulos et al., 2014), future studies in non-diabetic patients are warranted. Fourth, as noradrenergic subtype of PD is often associated with worse cognitive function (Ray Chaudhuri et al., 2023), PD patients with distinct autonomic dysfunction could have been enrolled in our study. Our study results may add an evidence to prior studies revealing preserved noradrenaline transporter binding in the locus coeruleus of the patients with tremor-predominant PD (Paulus and Jellinger, 1991, Kinnerup et al., 2021). However, future large-scale unbiased study is warranted to confirm this association between LF power, tremor severity, and cerebellothalamic circuit.

## 5 Conclusion

In our study, reduced LF power was associated with the diagnosis of PD, and preservation of LF power was associated with higher tremor severity and lower cerebellar cortex volume. These results indicate that LF power may be used as a biomarker for tremor-associated pathophysiology in PD. In addition, the wearable ECG device, with its capability for long-term monitoring, shows promise as a highly feasible and reliable tool for the early diagnosis of PD.

## Data Availability

The raw data supporting the conclusions of this article will be made available by the authors, without undue reservation.

## Footnotes

1
https://surfer.nmr.mgh.harvard.edu
