# Supplementary material for: Implications of heart rate variability measured using wearable electrocardiogram devices in diagnosing Parkinson’s disease and its association with neuroimaging biomarkers: a case-control study
Source: Front Aging Neurosci. 2025 May 12;17:1530240. doi: 10.3389/fnagi.2025.1530240 (PMC12104211; doi:10.3389/fnagi.2025.1530240)
Supplement: Supplementary file 1 [file Data_Sheet_1.docx]

Supplementary Table 1. Pairwise comparison of ROC curve models

|  | AUC difference | 95% CI of AUC difference | P-value |
| --- | --- | --- | --- |
| Model 1 – Model 2 | 0.006 | -0.149 – 0.161 | 0.941 |
| Model 1 – Model 3 | 0.050 | -0.047 – 0.147 | 0.311 |
| Model 1 – Model 4 | 0.065 | -0.102 – 0.231 | .0446 |
| Model 1 – Model 5 | 0.018 | -0.124 – 0.160 | 0.808 |
| Model 1 – Model 6 | -0.050 | -0.121 – 0.021 | 0.166 |
| Model 2 – Model 3 | 0.044 | -0.063 – 0.151 | 0.420 |
| Model 2 – Model 4 | 0.059 | -0.003 – 0.121 | 0.062 |
| Model 2 – Model 5 | 0.012 | -0.081 – 0.105 | 0.804 |
| Model 2 – Model 6 | -0.056 | -0.182 – 0.070 | 0.385 |
| Model 3 – Model 4 | 0.015 | -0.093 – 0.123 | 0.789 |
| Model 3 – Model 5 | -0.032 | -0.165 – 0.101 | 0.633 |
| Model 3 – Model 6 | -0.100 | -0.209 – 0.009 | 0.072 |
| Model 4 – Model 5 | -0.047 | -0.164 – 0.070 | 0.429 |
| Model 4 – Model 6 | -0.115 | -0.251 – 0.032 | 0.125 |
| Model 5 – Model 6 | -0.068 | -0.164 – 0.029 | 0.169 |

Model 1 includes age, sex, and HR_max_-HR_min_ as independent variables, and diagnosis of Parkinson’s disease (PD) as dependent variable. Model 2 includes age, sex, and SDNN as independent variables, and diagnosis of PD as dependent variable. Model 3 includes age, sex, and STD as independent variables, and diagnosis of PD as dependent variable. Model 4 includes age, sex, and RMSSD as independent variables, and diagnosis of PD as dependent variable. includes age, sex, and LF power as independent variables, and diagnosis of PD as dependent variable. includes age, sex, HR_max_-HR_min_, SDNN, STD, RMSSD, and LF power as independent variables, and diagnosis of PD as dependent variable. Pairwise comparison of ROC curves did not reveal any significant differences among 6 models.

Abbreviations: ROC, receiver operating curve; AUC, area under the curve; CI, confidence interval; HR_max_-HR_min_, difference between maximal heart rate and minimal heart rate; LF, low frequency; RMSSD, root mean square successive difference; SDNN, standard deviation of N-N intervals; STD, standard deviation of heartbeats

Supplementary Table 2. Association between LF power and tremor associated brain region volume

|  | Correlation coefficients | p-value |
| --- | --- | --- |
| Left thalamus | -0.528 | 0.052 |
| Right thalamus | -0.445 | 0.111 |
| Left cerebellar hemisphere | -0.598 | 0.024 |
| Right cerebellar hemisphere | -0.693 | 0.006 |

Age, sex, and intracranial volume were included as covariates in the Pearson partial correlation analysis.

Abbreviations: LF, low frequency.

Supplementary Figure 1.


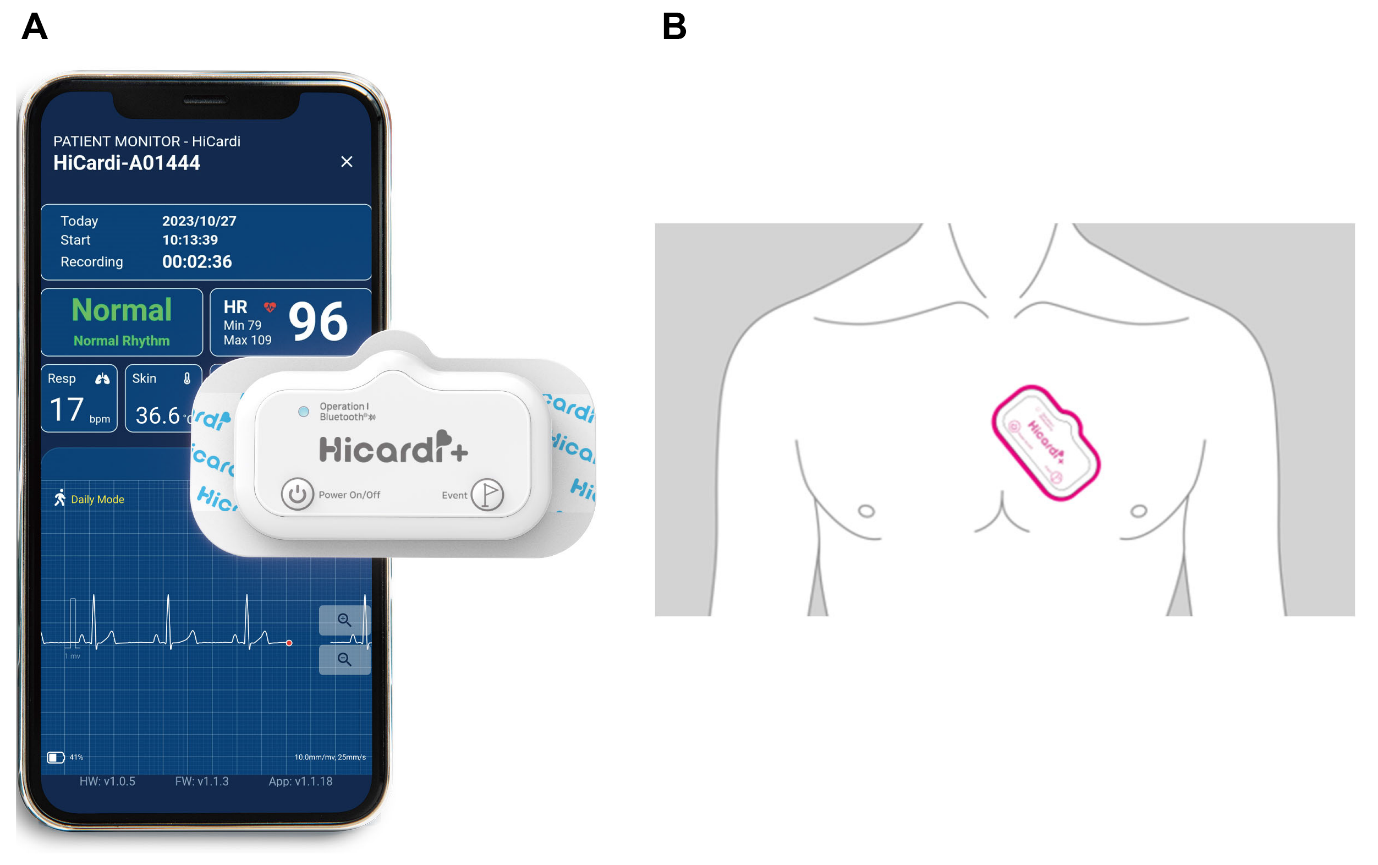


Medical devices for collecting heart rate variability in this study (A), and the attachment location of the device (B).
